# Immune Checkpoint Inhibitor-associated Pneumonitis: A Narrative Review

**DOI:** 10.5811/westjem.20305

**Published:** 2025-02-07

**Authors:** Chang Li, Saadia A. Faiz, Megan Boysen-Osborn, Ajay Sheshadri, Monica K. Wattana

**Affiliations:** * McGovern Medical School at University of Texas Health, Divisions of Pulmonary, Critical Care Medicine and Sleep Medicine, Houston, Texas; † The University of Texas MD Anderson Cancer Center, Department of Pulmonary Medicine, Houston, Texas; ‡ University of California Irvine School of Medicine, Department of Emergency Medicine, Irvine, California; § The University of Texas MD Anderson Cancer Center, Department of Emergency Medicine, Houston, Texas

## Abstract

Immune checkpoint inhibitors (ICI), such as pembrolizumab, nivolumab, durvalumab and ipilimumab, have significantly enhanced survival rates for multiple cancer types such as non-small cell lung cancer, melanoma, Hodgkin lymphoma, and breast cancer, and they have emerged as an adjunct or primary therapy for malignant disease. Approximately 40% of patients with cancer on ICI therapy experience side effects called immune-related adverse events (irAE). While not the most common, pulmonary toxicities can be rapidly progressive, potentially fatal, and pose a three-fold increased risk for requiring intensive care unit-level of care. Pneumonitis is a focal or diffuse inflammation of the lung parenchyma, and clinical manifestations may be highly variable. While the onset is generally observed 6–12 weeks after the initiation of therapy, drug toxicity can develop rapidly within days after the first infusion or many months into therapy. Pneumonitis symptoms can be subtle or non-specific; therefore, a thorough and systematic evaluation considering other possible etiologies is crucial. Moreover, extrapulmonary findings, such as skin lesions, colitis, or endocrinopathies, should raise suspicion for irAE as drug toxicity can affect multiple organs simultaneously. Due to the significant overlap of clinical features between ICI-associated pneumonitis and respiratory infections, it can be challenging to differentiate the two conditions based on clinical presentation alone. A multidisciplinary approach to management is recommended for the treatment of ICI-associated pneumonitis, and classification of severity helps to guide interventions. Treatment options in more severe cases include systemic immunosuppression. Given the increased use of ICIs and greater probability that patients with ICI-associated pneumonitis will be seen in the emergency department, we aimed to provide a comprehensive framework for the diagnosis and management. In addition, identifying potential challenges in diagnosis and/or other contributors of respiratory symptoms and radiographic manifestations is highlighted.

## INTRODUCTION

Patients with cancer frequently require care in emergency departments (ED) owing to acute presentations of malignant disease, cancer-associated complications, therapy-related adverse events, and/or other coexisting comorbidities. Fortunately, mortality has improved among many cancer types.^1^ ^,^ ^2^ In particular, immune checkpoint inhibitors (ICI) have significantly impacted survival rates, used alone or as supportive therapy to conventional cancer treatments.^3^ Given the efficacy of ICIs, it is likely that emergency physicians will see increasing numbers of cancer patients on ICIs in the years to come.^4^

Immune checkpoint inhibitors, such as pembrolizumab, nivolumab, and ipilimumab, work by blocking checkpoint protein-binding. This inhibitory signal removal allows T-cells to attack cancer cells. Approximately, 40% of patients on ICIs experience side effects called immune-related adverse events (irAEs).^5^ Patients with irAEs often present with subtle and non-specific symptoms that may mimic other diagnoses; therefore, detection of irAEs can be challenging. Furthermore, they can involve (almost) every organ system. Patients diagnosed with irAEs in the ED generally present with higher-grade toxicities, and 3.5% of patients with grade 3 irAEs require hospitalization and corticosteroid treatment.^6^ Delays in identification of irAEs may result in worsened prognosis and longer hospital lengths of stay.^6^ ^,^ ^7^

While toxicities of the pulmonary system are not the most common irAE, they occur in up to 10% of patients.^8^ When present, pulmonary toxicities can be rapidly progress; they are potentially fatal and associated with a substantially increased risk for requiring intensive care unit-ICU level care.^9^ ^,^ ^10^ Thus, prompt recognition of ICI-related pneumonitis is paramount. In this review we aimed to provide a review of the clinical presentation, risk factors, diagnostic approach, and management of pulmonary irAEs in the ED.

## CLINICAL PRESENTATION

**Figure 1.**
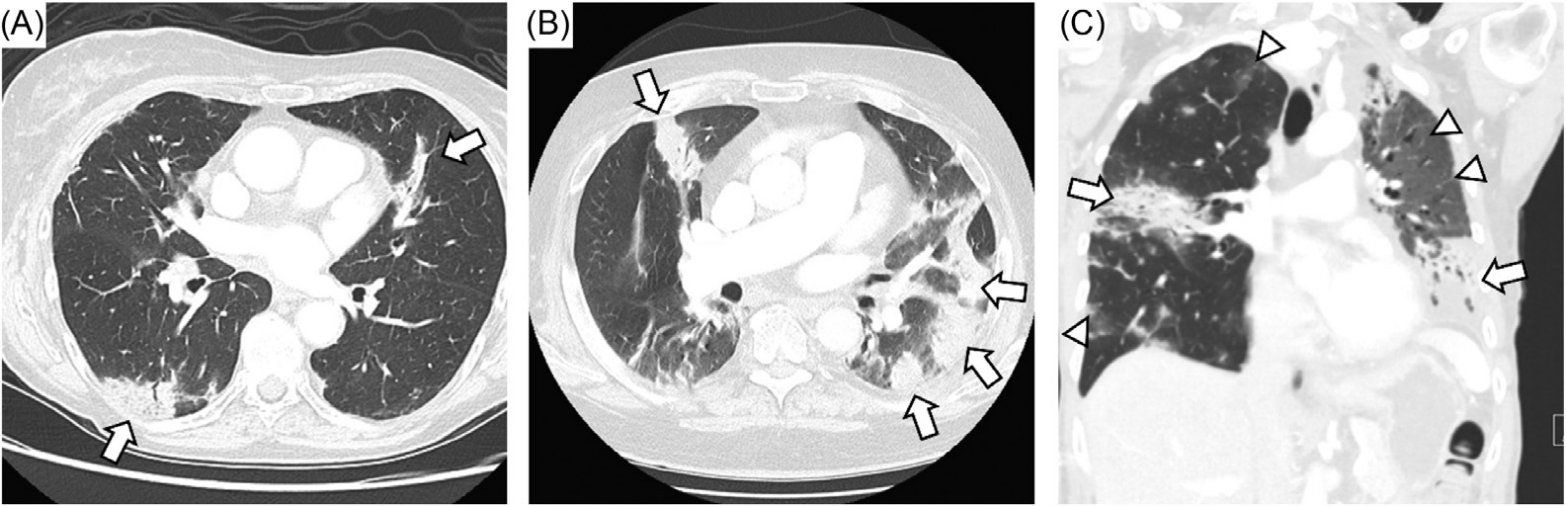
Representative case of immune checkpoint inhibitor- (ICI) associated pneumonitis. A) Elderly woman with melanoma treated with ICI presented with patchy bilateral consolidative opacities without any respiratory symptoms (Grade 1). B) Elderly man with melanoma affecting scalp underwent surgery followed by neoadjuvant ICI presented with persistent dry cough. Computed tomography of the chest (CT chest) revealed multifocal consolidative opacities. He underwent bronchoscopy with biopsy of lymph nodes and bronchoalveolar lavage without evidence of malignancy or infection. He was diagnosed with Grade 2 ICI-associated pneumonitis, and he improved with oral steroids. C) Middle-aged woman with triple negative breast cancer on ICI presented with cough and dyspnea with exertion not improved on outpatient oral steroid therapy. On physical exam she was noted to be tachypneic and hypoxic on room air. Coronal CT chest revealed consolidative opacities on the right and left along with ground-glass infiltrates on the left upper lobe. She was admitted and treated for Grade 3 ICI-associated pneumonitis with intravenous methylprednisolone (1 mg/kg) followed by infliximab. She improved and was discharged on prolonged steroid taper.

Pneumonitis is focal or diffuse inflammation of the lung parenchyma, and clinical manifestations may be highly variable.^8^ ^,^ ^11^ Onset of pneumonitis from ICIs is usually 6–12 weeks after the initiation of therapy, but drug toxicity can develop rapidly within days of the first infusion or many months into therapy.^8^ ^,^ ^11^ ^–^ ^14^ Shorter time to onset of irAEs is seen in patients with lung cancer compared to other types of malignancy, perhaps due to comorbid pulmonary disease, particularly underlying interstitial lung disease.^15^ The severity of symptoms associated with ICI pneumonitis can range from asymptomatic with only radiographic changes to life-threatening, fulminant respiratory failure (Figure 1). Common symptoms may include exertional dyspnea, cough, fatigue, and decreased activity tolerance; hypoxemia may present acutely or insidiously. Fever and/or chest pain, when present with other respiratory symptoms, should prompt a search for other etiologies, including pneumonia.

**Figure 2.**
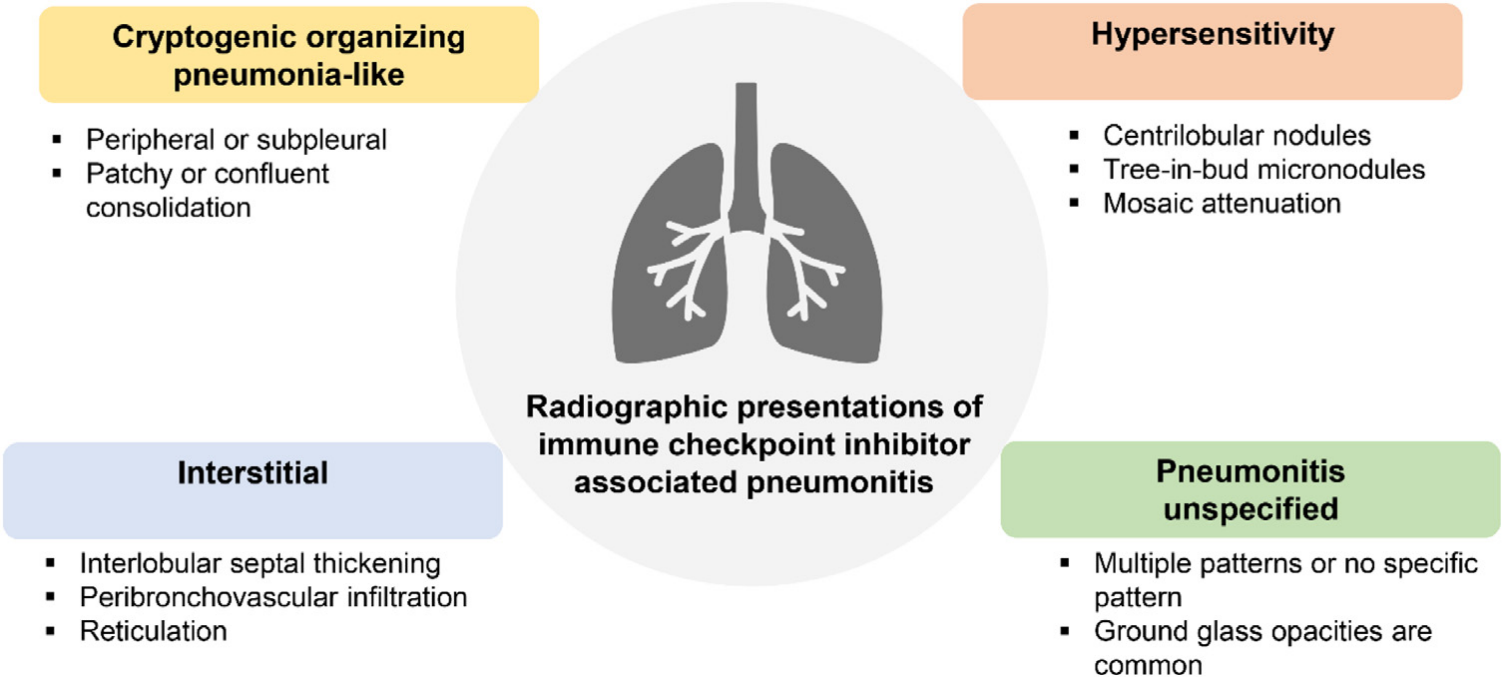
Radiographic manifestation of immune checkpoint inhibitor-associated pneumonitis.

Because the symptoms of pneumonitis can be subtle or non-specific, a thorough evaluation is crucial in reaching the correct diagnosis. Competing diagnoses, such as respiratory infections, cardiogenic pulmonary edema, disease progression of the underlying malignancy, and other drug-related complications must be considered. Moreover, extrapulmonary findings, such as skin lesions, colitis, or endocrine disorders, should raise the suspicion of irAEs, as drug toxicity can affect multiple organs simultaneously. Additional information from computed tomography (CT) of the chest (Figure 2) and bronchoscopy is usually incorporated to exclude alternative diagnoses.

Separate from pneumonitis, infusion reactions are adverse reactions unrelated to the mechanism of action of ICIs. Although relatively uncommon, they have been shown in 4% of patients treated with programmed cell death 1 (PD-1) or program death-ligand 1 (PD-L1) antibodies and in 2–6% of patients treated with ipilimumab (cytotoxic T-lymphocyte antigen or CTLA-4 inhibitor).^16^ ^,^ ^17^ The onset of symptoms can occur within any time frame during the infusion or up to one hour after the infusion. Symptoms include chest tightness, wheezing, rigors, rash, pruritus, tongue swelling, dizziness, tachycardia, hypotension/hypertension, or anaphylaxis. Infusion reactions are typically mild to moderate and usually resolve with the cessation of infusion and supportive care. However, severe reaction such as anaphylaxis can occur; therefore, premedication with glucocorticoids and antihistamine should be used when the patient has had prior reactions.^18^

## RISK FACTORS

The clinical profile of irAEs is dependent on the affected organ and the ICI agent used. For example, CTLA-4 agents are more likely to cause colitis and dermatitis than pneumonitis or thyroiditis.^19^ While the pathophysiology of ICI-related pneumonitis is not fully understood, potential risk factors have been reported and can be categorized as treatment-related, patient-related, and tumor-related factors, and the presence or absence of them can modify one’s risk of developing pneumonitis induced by the treatment.

### Treatment-related Factors

There are different degrees of pulmonary toxicity depending on whether ICIs are used as a single agent vs in combination with another ICI agent, targeted therapy, or radiation. In general, pneumonitis occurs more frequently in patients treated with PD-1 inhibitors, as compared to patients treated with PD-L1 inhibitors or with CTLA-4 inhibitors.^16^ ^,^ ^20^ ^,^ ^21^ Further, PD-1 and PD-L1 inhibitors cause pulmonary toxicity more often than CTLA-4 inhibitors.^22^ For example, in patients with advanced non-small cell lung cancer (NSCLC), a combination of PD-1 and CTLA-4 inhibitors (nivolumab/ipilimumab) resulted in a higher response rate and better progression-free survival time than those receiving chemotherapy alone.^23^ However, pneumonitis, particularly high-grade pneumonitis, occurred more often with combination therapy than ICI monotherapy or chemotherapy, suggesting a synergistic effect in inducing lung inflammation.^11^ ^,^ ^22^ ^,^ ^24^ Furthermore, the risk of pneumonitis may increase when ICIs are used in combination with epidermal growth factor receptor-tyrosine kinase inhibitors (EGFR-TKI) NSCLC. Specifically, patients treated with osimertinib (EGFR-TKI), followed by a PD-1 or a PD-L1 inhibitor, are at a high risk of pneumonitis.^25^ ^,^ ^26^

Pneumonitis after thoracic radiation is well documented, raising the concern of enhanced pulmonary toxicity with the combination of ICIs and radiation therapy.^27^ ^,^ ^28^ In general, radiation doses correlate with the risk of lung injury. An observational study illustrated that more patients developed ICI-related pneumonitis in the group that received curative intent radiotherapy than the group that received palliative intent radiotherapy.^29^ Other studies have noted that mean lung dose is a significant risk factor for pneumonitis, with or without ICI.^30^ ^,^ ^31^ Radiation-recall pneumonitis is an inflammatory reaction that occurs within previously irradiated fields following exposure to certain chemotherapy (taxanes, anthracyclines, alkylating agents, antimetabolites, or pyrimidine analogs) or other medications (tamoxifen, simvastatin, levofloxacin, or isoniazid), often months to years apart from previous radiation exposure.^32^ ^,^ ^33^ Radiation-recall pneumonitis induced by ICI agents has been reported in case reports.^34^ ^,^ ^35^ In contrast to the common radiographic patterns associated with ICI pneumonitis, radiation recall pneumonitis is generally confined to area of prior thoracic radiation.

Chemotherapy may also enhance one’s risk for ICI pneumonitis. The PACIFIC study demonstrated striking survival benefits with durvalumab (PD-L1 inhibitor) as adjuvant therapy after chemoradiation.^36^ However, a higher incidence of pneumonitis was also found in the durvalumab group (34%) compared to the placebo group (25%). Therefore, patients with advanced NSCLC treated with concurrent chemoradiation and ICIs are much more likely to develop pneumonitis than with concurrent chemoradiation alone. In general, higher radiation doses also increase the risk of lung injury.

### Patient-related Risk Factors

Pre-existing lung conditions, particularly interstitial lung disease (ILD), have been recognized as an independent risk factor for lung injury after ICI therapy.^11^ ^,^ ^13^ Patients with ILD were previously excluded from clinical trials due to concern of potential exacerbation with immunotherapy. Therefore, the efficacy and safety of ICI use in patients with underlying interstitial abnormalities has been an active area of interest. Multiple retrospective studies have demonstrated that patients with ILD who received ICI therapy were more likely to develop ICI pneumonitis. Patients with NSCLC have a higher rate of pre-existing ILD than other solid tumors, owing to the fact that both lung cancer and ILD are closely associated with smoking and other factors such as advanced age.^37^ Patients with NSCLC and pre-existing lung diseases including ILD and chronic obstructive lung disease (COPD), can have impaired survival once pneumonitis develops.^8^ The risk for pneumonitis may also be higher in patients with interstitial lung abnormalities without clinical ILD.^15^ Considering the association between ILD and lung cancer, ICI-related complications are a major concern in this patient population given the shifting paradigm favoring ICI therapy.

Additional patient-related risk factors to consider include autoimmune diseases and smoking. Retrospective studies showed that patients with autoimmune disease may have higher rates of immunotoxicity, including flares of their pre-existing autoimmune conditions and/or irAEs related to ICI therapy.^38^ ^,^ ^39^ In a multicenter cohort study, 71% of patients with autoimmune conditions, such as rheumatoid arthritis and psoriatic arthritis, were noted to have flares or irAEs, which were mostly manageable with glucocorticoids.^40^ Whether smoking is directly or indirectly linked to ICI-related pneumonitis is unclear, especially when considering the close connection between smoking, ILD, and lung malignancy. In one study, patients with lung cancer and tobacco exposure more than 50 years had higher incidence of all-grade pneumonitis.^41^

### Tumor-related Risk Factors

Certain tumor types and histology are at higher risk of ICI-related pneumonitis. One meta-analysis on clinical trials of ICI agents (PD-1, PD-L1, and CTLA-4) from 2003–2015 found that pneumonitis was more likely to occur in NSCLC and renal cell carcinoma as compared to melanoma.^42^ Another study reported higher rates of pneumonitis in patients with NSCLC treated with PD-1 antibody.^24^ Additionally, squamous cell carcinoma, a subtype of NSCLC that is typically found in patients with smoking history, was shown to be more associated with pneumonitis when compared to other subtypes of NSCLC.^43^ However, other studies have not demonstrated a link between NSCLC subtype and pneumonitis risk.^8^ This discrepancy may be because squamous cell cancer is more common in patients who smoke, and patients who smoke have a higher rate of pneumonitis that may be mediated by the presence of interstitial lung abnormalities or clinical ILD.

## DIAGNOSTIC APPROACH

**Figure 3.**
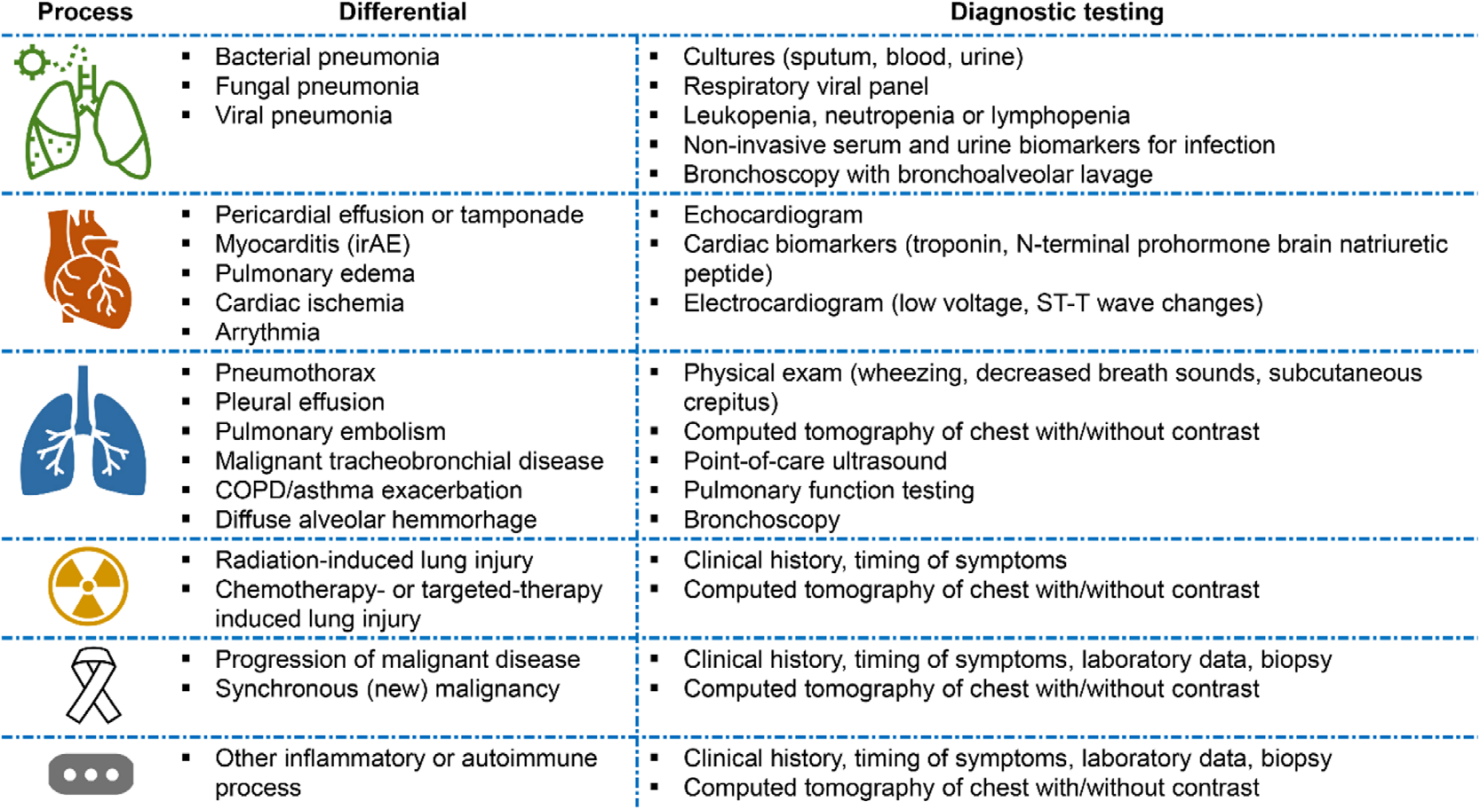
Differential diagnosis for immune checkpoint inhibitor-associated pneumonitis. *COPD*, chronic obstructive pulmonary disease; *irAE*, immune-related adverse events.

Evaluation of the cancer patient with respiratory symptoms, fever and/or hypoxia can be challenging, and a broad differential is needed (Figure 3). There are many other conditions that may be difficult to distinguish from ICI-associated pneumonitis or with which an irAE may coexist. Because the symptoms of pneumonitis can be subtle or non-specific, a thorough evaluation is crucial in reaching the correct diagnosis. Competing diagnoses, such as respiratory infections, cardiogenic pulmonary edema, disease progression of the underlying malignancy, and other irAE must be considered. As mentioned previously, extrapulmonary findings, such as skin lesions, colitis, or endocrine disorders, should raise the suspicion irAE as drug toxicity can affect multiple organs simultaneously.

**Figure 4.**
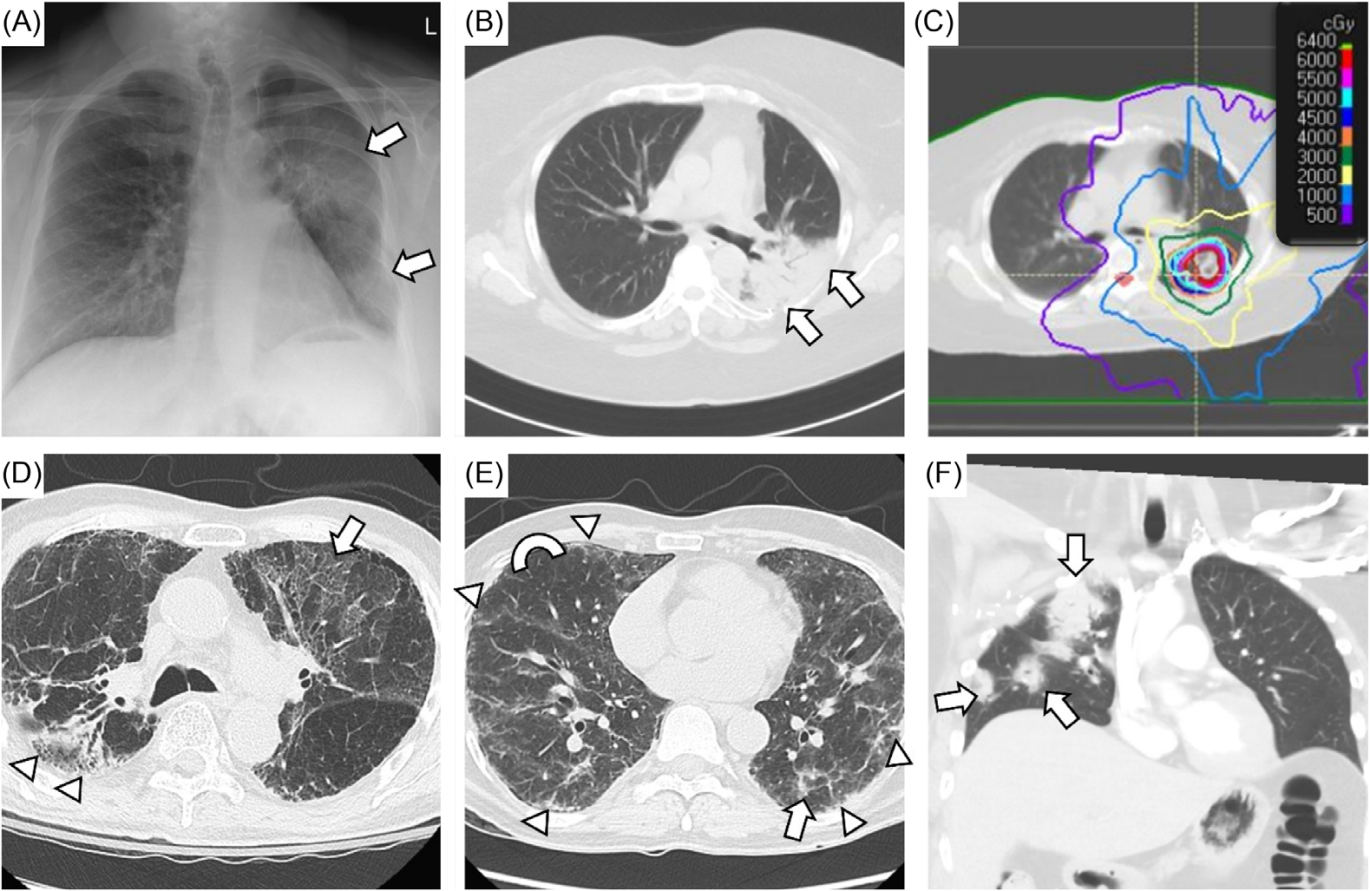
Challenging cases of possible immune checkpoint inhibitor (ICI) pneumonitis. A-C) Middle-aged woman with metastatic renal cell carcinoma treated with cabozatinib and nivolumab and radiation to the left upper lobe. She presented with cough and dyspnea with exertion (Grade 2) six months after radiation therapy was completed. Chest radiograph reveals new infiltrates in the left lung (arrows), and computed tomography of chest (CT chest) demonstrates dense consolidation in the left upper lobe with air bronchograms (B, arrows). Her infiltrates corresponded to radiation field (C). She was diagnosed with radiation-related lung injury and improved with oral steroid; however, ICI-associated pneumonitis could have also contributed. D) Elderly man with non-small cell lung cancer who underwent definitive chemoradiation therapy to the right upper lobe followed by pembrolizumab. He was hospitalized after a fall, and he was noted to have low oxygen saturations. CT chest revealed post-treatment changes in the right upper lobe (arrowheads) and bilateral ground-glass upper lobe infiltrates (arrow) in the setting of diffuse emphysematous changes. He was treated for chronic obstructive pulmonary disease (COPD) exacerbation with empiric antimicrobial therapy and oral steroids. His steroids were prolonged given lack of clinical improvement, so ICI-associated pneumonitis was also a concern. Bronchoscopy was not feasible due to significant oxygen requirement. E) Middle-aged man with papillary thyroid cancer who had undergone resection and treatment with carboplatin and paclitaxel. He was subsequently treated with pembrolizumab and presented with cough and dyspnea with exertion. He also had history of vocal cord dysfunction with paralyzed left vocal cord and aspiration risk. CT chest revealed diffuse peripheral and subpleural thickening (arrowheads), ground-glass opacities (arrow), and mosaic attenuation (semicircle showing contrast). He underwent bronchoscopy with lavage, and he was treated empirically for infection and with IV steroids for possible drug-related pneumonitis. Other potential etiologies included aspiration pneumonia, interstitial lung disease, and COPD exacerbation. F) Middle-aged woman with HER2-positive breast cancer treated with pembrolizumab hospitalized for fever, cough and dyspnea with exertion. Coronal CT chest reveals patchy opacities (arrows) affecting the right upper, middle, and lower lobes. Bronchoscopy was performed, and no obvious infection was found. She was treated with empiric antimicrobial therapy and iIV steroids for presumed ICI-associated pneumonitis. She was discharged on oral steroids therapy with *Pneumocystis jirovecii* prophylaxis.

Pneumonitis associated with ICI is a clinical diagnosis, and both malignant and infectious etiologies should be excluded.^44^ Physical exam findings can be normal or may include rhonchi or rales on auscultation. Unfortunately, there are no pathognomonic symptoms or radiographic findings that confirm ICI-associated pneumonitis; therefore, a systematic diagnostic approach is needed to exclude other clinical possibilities (Figure 4).^45^ ^,^ ^46^ Current guidelines recommend thorough evaluation including CT chest with (angiography if concern for pulmonary embolism) or without contrast and bronchoscopy to exclude alternative diagnoses. Laboratory tests may show leukocytosis and/or elevated inflammatory markers potentially supporting a diagnosis of irAE, but these are non-specific. Pneumonitis is graded based on radiographic and/or clinical severity (Table) and helps to direct further management.

Due to the significant overlap of clinical features between ICI-associated pneumonitis and respiratory infections, it can be challenging to differentiate the two conditions based on clinical presentation alone. Per American Society of Clinical Oncology guidelines, a thorough infectious workup, including nasal swab for respiratory viral pathogens, sputum culture, blood culture, and urine culture, is recommended for grade 2 and above pneumonitis. Patients receiving ICI agents are not necessarily at higher risk of infection. In a single-center study, patients with lung cancer treated with both ICI and chemotherapy had a similar rate of infection (15%) as the control group treated with chemotherapy alone (22%).^47^ However, patients treated with immunosuppressive agents for irAEs, such as corticosteroids or tumor necrosis factor (TNF) inhibitors, are at higher risk for opportunistic infection and tuberculosis reactivation. Overall, the incidence of infection in patients with lung cancer receiving ICI therapy ranges between 15–20%.^47^ ^,^ ^48^ The incidence of infection varies with different tumor types. Bacterial pneumonia is the most common type of infection and risk factors include diabetes, COPD, and neutropenia. Prior colonization or infection with *Pseudomonas aeroginosa* or recent exposure to parenteral antibiotics are indications to select antimicrobial agents targeting *Pseudomonas* species.^49^

## MANAGEMENT

A multidisciplinary approach to management is recommended for the treatment of ICI-associated pneumonitis, and classification of severity (Table 1) helps to guide interventions.^50^ ^–^ ^52^ It is imperative to involve infectious, pulmonary, and/or oncologic consultants early to determine the most appropriate treatment, especially for complex cases with multiple etiologies. Treatment options generally consist of temporary ICI cessation with regular clinical reassessment, and in more symptomatic cases, systemic immunosuppression may be required.^53^ For grade 1 (asymptomatic) pneumonitis, ICI agents may be withheld when there is radiographic evidence of pneumonitis progression, but in many cases the ICI agent can be continued with close clinical and radiologic reassessment for development of respiratory symptoms. If the patient remains asymptomatic, steroids are not typically administered. For grade 2 (mildly to moderately symptomatic) pneumonitis, steroids, such as prednisone or methylprednisolone administrated orally or intravenously, are given at 1–2 milligrams per kilogram per day (mg/kg/d) following infectious workup to exclude other potential etiologies. If symptoms do not improve after 48–72 hours, a higher dose of steroids should be considered. Mild grade 2 cases can be treated with the lower dose of 1 mg/kg if the response to treatment is rapid. For grade 3 or higher (severely symptomatic), prednisone or methylprednisolone are given at 1–2 mg/kg/d with close monitoring.^50^ If no clinical improvement occurs within 48–72 hours, other immunomodulators (discussed in the **Special Situations** section below) should be considered to prevent further respiratory decompensation. It is recommended to obtain evaluation from consultants before administration of immunosuppressants, such as steroids, as these agents can have large impact on the overall clinical outcome. In general, cases of pneumonitis grade 3 and higher result in permanent ICI discontinuation. Dosing and tapering course of steroids for ICI pneumonitis are largely extrapolated from treatment guidelines for hypersensitivity pneumonitis and cryptogenic organizing pneumonia.^54^ ^,^ ^55^ Current guidelines recommend a short corticosteroid taper over 4–6 weeks. However, retrospective studies have shown that pneumonitis may recur after improvement of symptoms or persist without improvement despite steroid treatment. Shorter courses of therapy may result in a higher chance of recurrence, but optimal steroid taper lengths have not been studied.

**Table.** Common terminology criteria for adverse events for immune checkpoint inhibitor-associated pneumonitis.^46^

Grading	Symptoms	Number of lobes involved (on CT)	OR	Percentage of lung parenchyma involved (on CT)
Grade 1 – mild	Asymptomatic	One		<25%
Grade 2 – moderate	Symptomatic	More than one		25–50%
Grade 3 – severe	Severe symptoms	All lobes		>50%
Grade 4 – life-threatening	Life-threatening respiratory failure	All lobes		>50%

*CT*, computed tomography.

Empiric antibiotics in patients presenting with respiratory symptoms while receiving ICI therapy is reasonable while further investigation is underway. One caveat is that the human microbiota plays an important role in the responses to cancer therapy.^56^ Antimicrobial use is known to alter the gut flora and has been shown with associated negative outcomes in patients receiving ICI therapy.^57^ Therefore, the appropriate and judicious use of antibiotics should be considered while infectious workup is carried out.

## SPECIAL SITUATIONS

Steroid refractory ICI-associated pneumonitis is characterized by a lack of improvement, typically, after 48 hours of corticosteroid treatment. Patients who develop steroid refractory pneumonitis tend to have worse clinical outcomes due to infectious complications or pneumonitis itself. When corticosteroids are ineffective in treating ICI pneumonitis, further immunomodulation may be required. Treatment guidelines suggest treating with agents such as intravenous immunoglobulin, anti-TNF agents, mycophenolate, or cyclophosphamide. However, data on the use of these agents is limited and mostly derived from case series or reports.^58^ In these studies, although some patients achieved clinical improvement with the addition of immunomodulators, the overall outcome was mostly poor.^59^ ^,^ ^60^ The choice of selecting these immunomodulators in treating steroid refractory ICI pneumonitis depends on the patient’s comorbidities and the clinician’s or the center’s experience. Of note, a negative interferon-gamma release assay, such as QuantiFERON, is often obtained before initiating anti-TNF agents due to the risk of tuberculosis reactivation. However, given that anti-TNF agents are typically given as 1 or 2 doses instead of long-term therapy, the short-term benefit of treating severe pneumonitis usually greatly outweighs any risk of reactivating indolent infections.

### Reintroduction of ICI Therapy After Pneumonitis

In general, patients who develop grade 2 pneumonitis and have recovered (ie, return to grade 1 pneumonitis), should be considered as eligible for reintroduction of ICI therapy. Only a few studies have assessed the rate of recurrent pneumonitis after ICI reintroduction. In a cohort of 107 patients who developed pneumonitis, 45 underwent re-challenge and of these, nine (20%) developed recurrent pneumonitis while 11 (24%) developed a different irAE.^61^ In a pharmacovigilance study including 452 irAEs occurring with ICI reintroduction in which recurrence status was verifiable, pneumonitis, colitis, and hepatitis were associated with an increased risk of recurrent irAE in adjusted analyses.^62^ While pneumonitis grade 3 and higher generally precludes ICI reintroduction, successful re-challenge has been reported.^63^ In general, these cases are rare, and ICI reintroduction in this scenario requires that the benefit with ICI clearly outweighs the high risk of recurrent and possibly severe pneumonitis.

### Steroid-dependent Pneumonitis

In some cases, pneumonitis does not resolve despite adequate corticosteroid therapy. In one form, Naidoo and associates have suggested an entity of chronic pneumonitis defined as a) pneumonitis that persists or worsens with steroid tapering; and b) requires more than 12 weeks of immunosuppression after ICI discontinuation.^14^ ^,^ ^64^ Two percent of patients with NSCLC and melanoma treated with anti-PD-L1 agents develop chronic ICI-associated pneumonitis.^13^ Steroid-dependent pneumonitis is a sub-type where pneumonitis recurs without some form of immunosuppression. There is little to guide the treatment of this form of pneumonitis, and uncertainty exists about the optimal non-steroidal immunosuppression, length of immunosuppression, cadence of steroid taper, and cancer outcomes in this scenario. While this form of pneumonitis rarely occurs, strategies can include treatment with low-dose steroid therapy or use of other immunomodulators such as mycophenolate mofetil before eventual attempting to taper.

## CONCLUSION

While immune checkpoint inhibitor-associated pneumonitis is less common than other adverse effects from ICIs, the potentially fatal consequences if missed makes diagnosis and prompt management by emergency physicians crucial. Associated risk factors are patient, tumor, and/or treatment related. Maintaining a high index of suspicion is important when evaluating patients with a history of ICI treatment presenting with respiratory symptoms. Workup in the ED involves imaging and lab work to rule out competing diagnosis such as infection and cardiac etiologies. Severity of ICI-pneumonitis is based on a grading system that considers clinical and radiographic findings; once suspected, prompt collaboration with oncologists and specialists is ideal, as treatment involves the initiation of high-dose steroids in the ED and possible cessation of ICI treatment. The integral role of the emergency physician in the timely diagnosis and management of ICI-associated pneumonitis is vital to improve patient outcomes.
